# TAPO in first‐line osimertinib therapy and continuation of osimertinib

**DOI:** 10.1111/1759-7714.14782

**Published:** 2022-12-28

**Authors:** Chihiro Mimura, Kazumi Kaneshiro, Shodai Fujimoto, Ryota Dokuni, Natsuhiko Iwamoto, Kanoko Matsumura, Yukihisa Hatakeyama, Yuko Kono, Motoko Tachihara

**Affiliations:** ^1^ Division of Respiratory Medicine, Department of Internal Medicine Kobe University Graduate School of Medicine Kobe Japan; ^2^ Department of Respiratory Medicine Kita‐Harima Medical Center Ono‐City Japan; ^3^ Department of Respiratory Medicine Akashi Medical Center Akashi Japan; ^4^ Department of Respiratory Medicine Hyogo Prefectural Awaji Medical Center Sumoto Japan; ^5^ Department of Respiratory Medicine Takatsuki General Hospital Osaka Japan

**Keywords:** interstitial lung disease, lung cancer, NSCLC, transient asymptomatic pulmonary opacity (TAPO)

## Abstract

**Background:**

Osimertinib is associated with a relatively high frequency of drug‐induced interstitial lung disease (D‐ILD), and transient asymptomatic pulmonary opacities (TAPO) have been reported to occur during osimertinib administration. The frequency of TAPO during first‐line treatment and the pros and cons of osimertinib continuation is unknown.

**Methods:**

This was a multicenter, retrospective study. The purpose of this study was to research the frequency of TAPO and to evaluate osimertinib continuation in first‐line therapy. We also evaluated progression‐free survival (PFS) including subgroup analysis.

**Results:**

From August 2018 to December 2020, 133 patients were enrolled into the study. The median observation period was 23.2 months (0.3–48.3 months). Thirty patients (22.6%) experienced D‐ILD events, including 16 patients (12.1%) with CTCAE grade 1, five patients (3.8%) with grade 2, and nine patients (6.7%) with grade 3 and above D‐ILD. Among the patients with grade 1 D‐ILD, 11 cases (8.3%) of TAPO were observed, and all patients succeeded in osimertinib continuation. The TAPO images were characterized by localized patchy opacities (73%). The median PFS was 22.6 months (95% confidence interval [CI]: 17.8–28.7 months). Patients with TAPO had a significantly longer PFS than patients with non‐TAPO D‐ILD in the multivariate analysis.

**Conclusions:**

This study showed that grade 1 D‐ILD might include TAPO and that patients with TAPO might have good PFS. We need to consider the possibility of osimertinib continuation when lung opacities appear.

## Graphical abstract

TAPOs were transient and asymptomatic lung opacities appeared during osimertinib administration. The frequency of TAPO in first‐line osimertinib therapy was 8.3% in this study. Patients who develop TAPO may be able to continue osimertinib administration and may have good PFS.

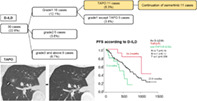

## INTRODUCTION

Lung cancer is the most common fatal disease according to Global Cancer Statistics.^1^ Adenocarcinoma is the most common type of lung cancer, and the frequency of *EGFR* mutation‐positive lung cancer is particularly high among the Asian population.^2^ Osimertinib is the standard first‐line therapy for patients diagnosed with advanced stage non‐small cell lung cancer (NSCLC) that is positive for *EGFR* mutations.^3^ Osimertinib has been shown to significantly prolong progression‐free survival (PFS) compared to first‐generation EGFR‐TKIs^4^ and significantly prolong overall survival (OS). However, a post hoc analysis of OS in Japanese patients showed no OS prolongation after osimertinib therapy, with a median OS of 39.3 months versus no OS in the control group (gefitinib).^5^ One reason for this finding may the discontinuation of osimertinib administration due to drug‐induced interstitial pneumonia (D‐ILD).

D‐ILD is one of the notable adverse events related to EGFR‐TKI use and has been reported to be more frequent in the Asian population,^6^ with a prevalence of approximately 12% among Japanese individuals.^7^ D‐ILD led to the discontinuation of osimertinib in 25.6% of patients in a previous study,^8^ and may also affect post‐osimertinib treatment.

On the other hand, transient asymptomatic pulmonary opacities (TAPO) have been reported to occur during osimertinib treatment.^9^ TAPO occurs in approximately 35% of patients,^10^ and most have localized opacities that resolve spontaneously. D‐ILD caused by osimertinib can be severe in some cases, but there have been reports of rechallenge osimertinib administration after the onset of D‐ILD.^11^, ^12^, ^13^, ^14^ It is possible that TAPO is among the D‐ILD cases diagnosed as grade 1, but there is no consensus regarding whether osimertinib can be continued when lung opacities appear.

In the present study, we aimed to research the frequency of TAPO as a first‐line treatment and whether osimertinib administration could be continued in the presence of pulmonary opacities.

## METHODS

### Study design and patients

This was a multicenter observational study. We retrospectively enrolled patients diagnosed with advanced NSCLC who were *EGFR* mutation‐positive and received osimertinib administration as a first‐line therapy from August 21, 2018, to December 31, 2020, at Kobe University Respiratory Medicine Group. The exclusion criteria were patients who were asked not to participate in this study based on publicly available information.

Data such as age, sex, Eastern Cooperative Oncology Group Performance Status (ECOG PS), smoking history, clinical stage based on the eighth edition of the TNM classification, brain metastasis, type of *EGFR* mutation, KL‐6, and programmed death‐1 (PD‐L1) expression were collected from the medical records of patients.

Consent was obtained from retrospectively enrolled patients by applying the opt‐out method. This study was approved by the Kobe University Ethics Committee (210111) on July 16, 2021, and by all participating institutions. This was conducted following the declaration of Helsinki. This study was registered in the University Medical Hospital Information Network in Japan (UMINCTR registration no. UMIN000044863, registered: July 19, 2021).

### CT imaging evaluation

We collected all CT images during osimertinib administration. All CT images were evaluated by two pulmonologists.

The frequency of D‐ILD was evaluated based on the Common Terminology Criteria for Adverse Events (CTCAE) version 5.0. TAPO was defined as a transient and asymptomatic opacity and was included in grade 1 D‐ILD. We also examined the time from appearance to disappearance of the lung opacities in cases with TAPO.

### Outcomes

The primary outcome was the frequency of TAPO and continuation of osimertinib administration. The secondary outcomes were PFS, including subgroup analysis such as PS, sex, smoking status, and *EGFR* mutation type, OS, time to treatment failure (TTF), and PFS2.

### Statistical analysis

Overall survival was defined as the time from the date osimertinib was started to the date of last confirmed survival in the medical record. Patients who were alive were terminated from the study on the date of last confirmed survival. In untraceable cases, the last date of confirmed survival before the loss of follow‐up was considered the cutoff date. TTF was defined as the time from the day osimertinib was started to the day that treatment was interrupted for any reason, including progression of the underlying disease, adverse effects of treatment, and any death. PFS was defined as the time from the date of osimertinib administration initiation until tumor progression (PD), clinical examination or death. PFS2 was defined as the time from the date of osimertinib administration initiation to the date when PD was confirmed in the second line of therapy after osimertinib treatment was completed. Treatment response was evaluated according to Response Evaluation Criteria in Solid Tumors (RECIST) version 1.1. Response to therapy was assessed by the treating physician.

The Kaplan–Meier (KM) method and log‐rank test were used for survival analysis. Cox proportional hazard models were used to evaluate the hazard ratios for factors. All statistical analyses were performed using EZR version 1.55 (Saitama Medical Center, Jichi Medical University, Saitama, Japan), a graphical user interfaces for R (The R Foundation for Statistical Computing, Vienna, Austria, version 3.6.3)^15^ The PFS, OS, and TTF were estimated using the KM method. They were compared using the log‐rank test. *p* < 0.05 was considered a statistically significant difference for all analyses.

## RESULTS

### Patient characteristics

**FIGURE 1:**
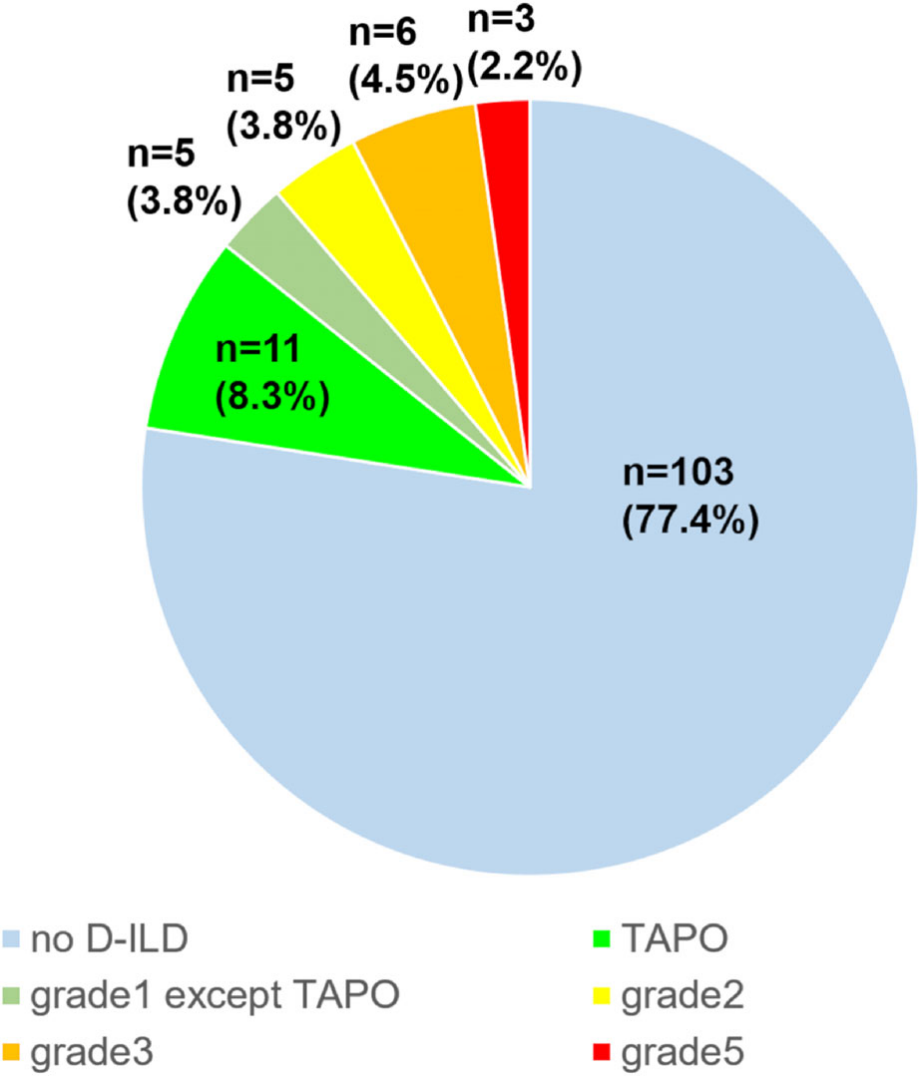
Frequency of drug‐induced interstitial lung disease (D‐ILD). The pie chart shows frequency of D‐ILD during osimertinib administration. Grade 1 D‐ILD was classified into transient asymptomatic pulmonary opacity (TAPO) and other than TAPO

A total of 133 patients were enrolled in this study. The patient characteristics are shown in Figure 1, Table 1. The median age was 73.5 (range 40–90) years, and females accounted for 64% (*n* = 85). More than two‐thirds of patients were nonsmokers (69%). The majority of patients were PS0 and 1. According to *EGFR* mutation type, 67 patients (50.4%) harbored exon 19 deletion, 59 patients (44.4%) harbored a point mutation in exon 21 resulting in L858R substitution, one patient (0.8%) harbored both exon 19 deletion and the L858R mutation, and six patients (4.5%) harbored minor mutations. The median observation period was 23.2 months (0.3–48.3 months).

**TABLE 1:** Patient characteristics

		All patients
Characteristics		(*n* = 133)
Age (years)		73.5
median		(40–90)
Sex	Male	48
	Female	85
Smoking status	Current or former	41
	Never	92
ECOG PS	0–1	103
	2–4	30
Stage	III	8
	IV	90
	Recurrence	35
Brain metastasis	Yes	38
	No	95
KL‐6 (U/ml)		366
median		(139–5915)
Mutation type	Exon 19 del	67
	L858R	59
	Exon 19 del/L858R	1
	Uncommon	6
PD‐L1 status (TPS)	<1%	45
	1%–49%	44
	>50%	18
	Unknown	26

Abbreviations: ECOG‐PS, Eastern Cooperative Oncology Group performance status; PD‐L1, programmed cell death ligand 1; TPS, tumor proportion score.

### Frequency of ILD, including TAPO and osimertinib continuation

Of the 133 patients, 30 patients (22.6%) experienced D‐ILD events. Sixteen patients (12.1%) had CTCAE grade 1, five patients (3.8%) had CTCAE grade 2, and nine patients (6.7%) had CTCAE grade 3 and above. Among the patients with grade 1 D‐ILD, 11 cases (8.3%) of TAPO were observed, and all patients succeeded in continuation of osimertinib. Osimertinib administration was discontinued among patients with D‐ILDs other than TAPO except for one case, wherein the patient resumed osimertinib administration voluntarily. Twelve patients required steroids for D‐ILD.

### TAPO characteristics

**FIGURE 2:**
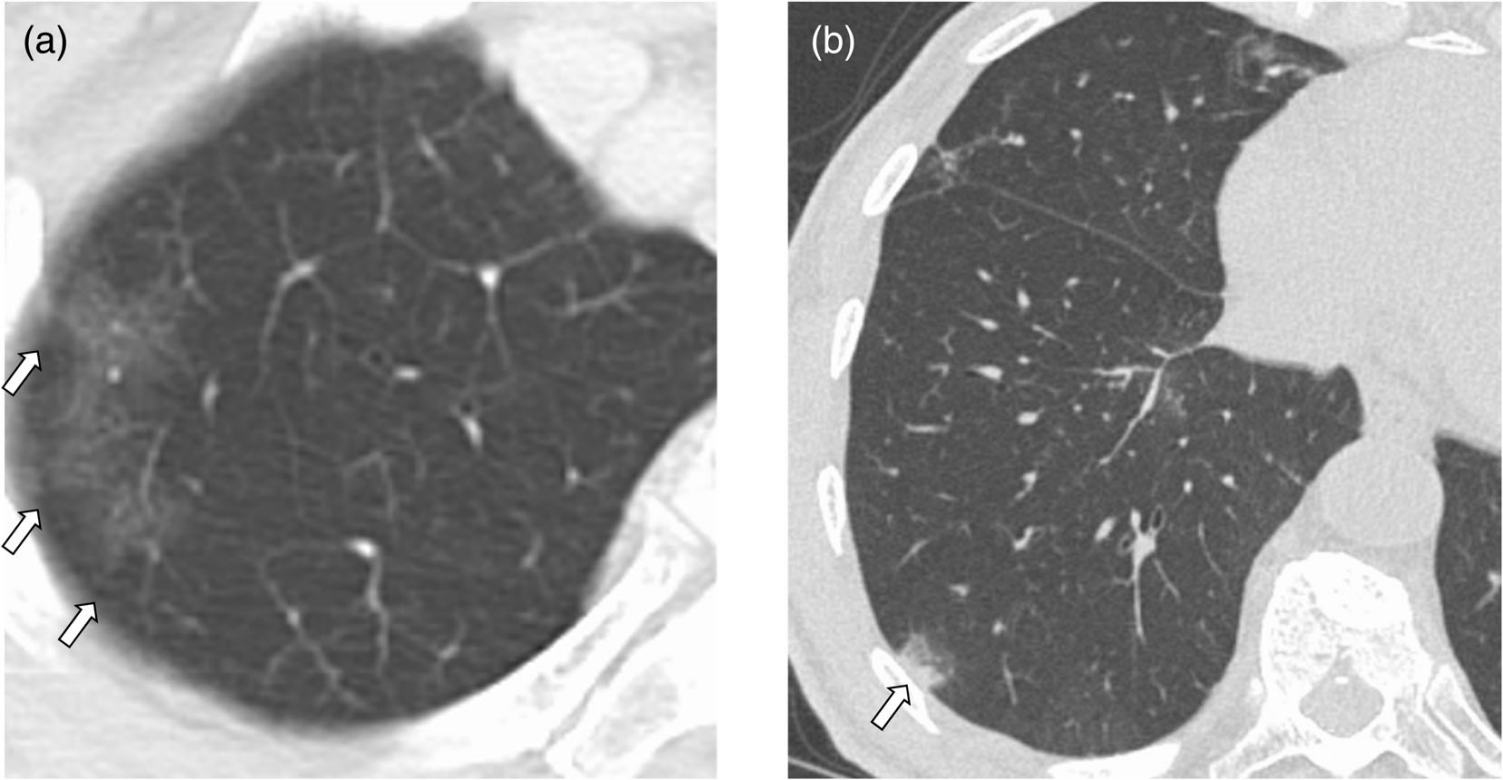
Shows a transient asymptomatic pulmonary opacities pattern. (a) Ground‐glass opacity, (b) localized patchy opacity

**FIGURE 3:**
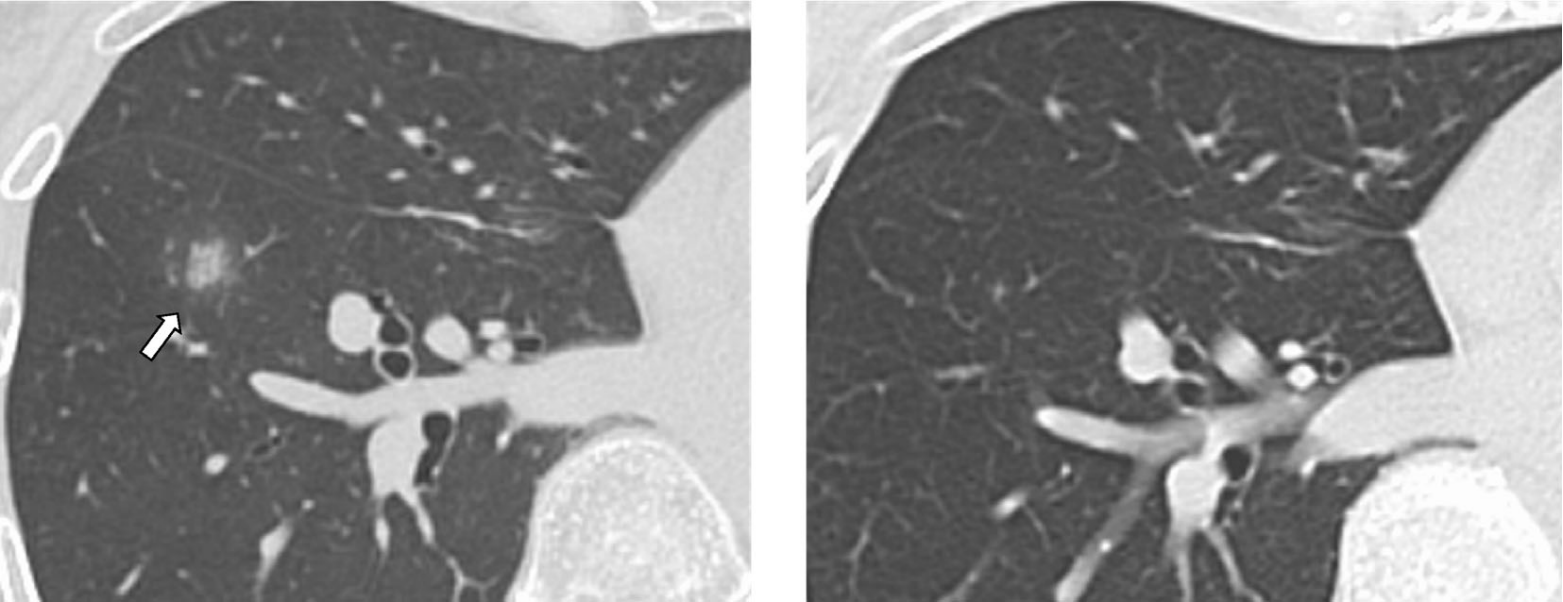
Transient asymptomatic pulmonary opacity (TAPO) from onset to disappearance. During the period of osimertinib administration, a patchy opacity, as indicated by the arrow, was observed in the right lower lobe, which disappeared after 2 months

Figure 2 shows TAPO images. The majority of TAPO images showed localized patchy opacities (73%). Some cases showed ground‐glass opacities (18%). All patients were asymptomatic at TAPO development. Patients with grade 1 D‐ILD except TAPO were also asymptomatic but had diffuse ground‐glass opacities. KL‐6 was not elevated in all patients, and eosinophils were elevated in only one patient. The median time to the onset of TAPO was 27 weeks (range: 5.7–115.9 weeks), and the median time to the disappearance of TAPO was 17 weeks (range: 8–36.4 weeks) (Figure 3). No recurrence of TAPO was observed in all cases. The characteristics of patients with TAPO and with non‐TAPO D‐ILD are shown in Table 2.

**TABLE 2:** Characteristics of patient with D‐ILD

Factor		TAPO (*n* = 11)	Non‐TAPO D‐ILD (*n* = 19)
Age (years)		77	78
median		(60–83)	(40–85)
Sex	Male	5	9
	Female	6	10
Smoking status	Current or former	2	7
	Never	9	12
ECOG PS	0–1	1	4
	2–4	10	15
KL‐6 (U/ml)		566.5	431
median		(237–4443)	(155–941)
Mutation type	Exon 19 del	8	12
	L858R	3	7
PD‐L1 status (TPS)	<1%	5	7
	1%–49%	4	3
	>50%	2	4
	Unknown	0	5

Abbreviations: D‐ILD, drug‐induced interstitial lung disease; ECOG‐PS, Eastern Cooperative Oncology Group performance status; PD‐L1, programmed cell death ligand 1; TAPO, transient asymptomatic pulmonary opacity; TPS, tumor proportion score.

### PFS including subgroup analysis, OS, and TTF

The median PFS was 22.6 months (95% confidence interval [CI]: 17.8–28.7 months). The PFS of patients with TAPO was significantly longer than that of patients with non‐TAPO D‐ILD, (34.2 vs. 13 months, hazard ratio 6.27, 95% CI: 1.7–23.1, *p* < 0.01.) This result was confirmed in the multivariate analysis (Table 3). On the other hand, there was no difference between patients without D‐ILD and with TAPO or between patients without D‐ILD and with non‐TAPO D‐ILD.

**TABLE 3:** . Cox proportional‐hazard models for progression‐free survival

		Univarate analysis		Multivariate analysis	
Factor		HR (95% CI)	*p*‐value	HR (95% CI)	*p*‐value
D‐ILD	TAPO	Reference		Reference	
	No D‐ILD	3.07	0.06	2.8	0.08
		(0.96–9.8)		(0.86–9.04)	
	non‐TAPO D‐ILD	6.27	0.006	6.06	0.007
		(1.7–23.1)		(1.64–22.5)	
PS	0–1	Reference		Reference	
	2–4	1.81	0.04	1.88	0.04
		(1.03–3.17)		(1.04–3.42)	
Sex	Male	Reference		Reference	
	Female	0.97	0.9	0.83	0.6
		(0.6–1.59)		(0.41–1.68)	
Smoking status	Never	Reference		Reference	
	Current or former	0.96	0.88	0.92	0.82
		(0.58–1.6)		(0.46–1.84)	
Mutation type	Exon 19 del	Reference		Reference	
	L858R	1.16	0.56	1.09	0.74
		(0.71–1.88)		(0.65–1.81)	
	Uncommon	1.09	0.89	0.99	0.99
		(0.3–3.58)		(0.29–3.37)	

Abbreviations: CI, confidence interval; D‐ILD, drug‐induced interstitial lung disease; HR, hazard ratio; TAPO, transient asymptomatic pulmonary opacity.

Of the 30 patients in which osimertinib administration due to D‐ILD was discontinued, nine (30%) received second‐line treatment. The severity of the D‐ILD among the nine patients was as follows: two patients with TAPO, three patients with grade 1 D‐ILD that was not TAPO, one patient with grade 2 D‐ILD, and three patients with grade 3 D‐ILD.

Patients with good PS had a significantly longer PFS than those with poor PS (23.3 vs. 11.9 months, hazard ratio 1.81, 95% CI: 1.03–3.17, *p* < 0.05). This result was also confirmed in the multivariate analysis. Another factor, sex, smoking status, and mutation type had no significant effect on PFS (Table 3).

**FIGURE 4:**
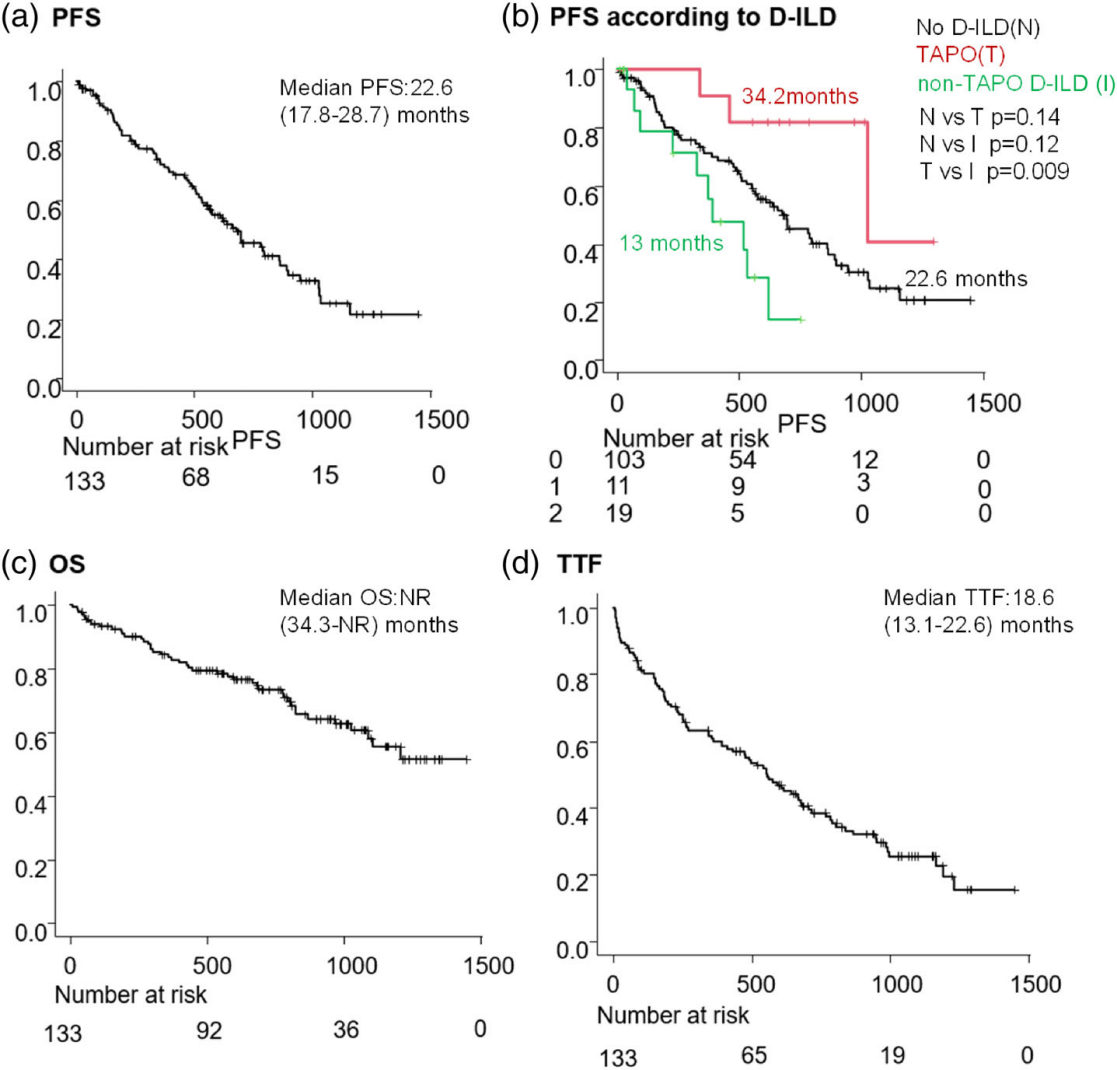
Osimertinib efficacy. (a) Kaplan–Meier curves of progression‐free survival (PFS). (b) Kaplan–Meier curves of PFS according to drug‐induced interstitial lung disease (D‐ILD). (c) Kaplan–Meier curves of overall survival (OS). (d) Kaplan–Meier curves of time to treatment failure (TTF). NR, not reached

During follow‐up, the median OS was not reached (Figure 4c). The median TTF was 18.6 months (95% CI: 13.1–22.6 months) (Figure 4d).

## DISCUSSION

Osimertinib is associated with a relatively high incidence of D‐ILD.^7^ In this study, the incidence of D‐ILD was 22.6%, similar to a previously reported rate.^16^ The prevalence of grade 3 or higher D‐ILD was 6.7%, which indicates a serious side effect. On the other hand, TAPO was identified in 8.3% of all cases, which is lower than a previously reported rate.^9^ Since TAPO is a transient and asymptomatic opacity, it is difficult to identify unless a chest CT scan is performed at the time of TAPO appearance. This was a retrospective observational study and may have underestimated the number of patients with TAPO due to, the fact that the interval between chest CT scans varied.

The cause of TAPO is unknown, but there have been no previous reports of TAPO with other EGFR‐TKIs. The fact that mechanism of action of osimertinib is different from that of other EGFR‐TKIs may be involved.^17^ Some D‐ILDs caused by molecularly‐targeted drugs are unique to each of them. For example, everolimus, an mTOR inhibitor, is associated with a relatively high frequency of D‐ILD^18^ but can be continued in grade 1 patients. Bortezomib has also been reported to cause lung injury, such as increased vascular permeability.^19^ Brigatinib, an ALK‐TKI, is known to cause lung opacities early in the course of treatment, known as early‐onset pulmonary events (EOPEs).^20^ It is very important to know the characteristics of D‐ILDs that could be caused by each drug.

With regard to imaging findings, previous reports have reported that idiopathic organizing pneumonia and eosinophilic pneumonia patterns are often the main features.^9^ In our study, localized patchy opacities accounted for 73% of the cases judged, but in some cases, ground‐glass opacities or infiltrated opacities were observed. We believe that localized patchy opacities were similar to those of previously reported simple eosinophilic pneumonia (SEP). In this study, we were unable to evaluate the eosinophils and perform bronchoscopy, so it was difficult to diagnose SEP as previously reported. However, in one case, eosinophils were elevated when the lung opacities appeared and decreased after the opacities disappeared, which may have been a diagnosis of SEP.

In this study, TAPO was often found localized within the same lung lobe, but there was no frequent lobes or laterality. The time of onset was also unclear, so it was sometimes difficult to determine whether a patient had TAPO based on imaging findings and onset time alone. Some patients may have been judged to have D‐ILD other than TAPO or worsening of their current disease, and osimertinib administration may have been discontinued, even if they were asymptomatic at the time of appearance of lung opacities. After osimertinib was readministered after the onset of D‐ILD, 17.9% of the patients again had D‐ILD.^21^ On the other hand, there have been reports that osimertinib could be continued in patients with TAPO.^22^, ^23^ We carefully continued osimertinib administration in patients with asymptomatic, localized opacities that were considered TAPO. In some cases, the opacities were so minor that the attending physician was unaware of the appearance of the opacities and continued osimertinib administration. Of the 11 patients who developed TAPO, two were discontinued due to adverse events other than D‐ILD, while the remaining nine patients still received osimertinib administration. Although careful judgment is needed, it may be possible to consider the possibility of TAPO and resume osimertinib administration.

Subgroup analysis of PFS was significantly longer in patients with TAPO than in those with D‐ILD except TAPO. One reason for this may be due to the discontinuation of osimertinib in D‐ILD cases. A previous report^16^ found no significant difference in PFS between TAPO and no D‐ILD cases. This study showed that TAPO tended to have better PFS than those with no D‐ILDs although the number of cases was small. The reason for this was unclear, but the onset of TAPO might contribute to good PFS.

The PFS was significantly shorter in patients with poor PS. Gefitinib is recommended for EGFR‐positive lung cancer patients with poor PS.^24^ However, the efficacy of osimertinib in patients with poor PS has also been reported in T790 M mutation‐positive lung cancer.^25^ Therefore, it is necessary to consider treatment in each case. No significant differences in PFS were observed for sex, smoking status, or mutation subtype, but the sample size was small, and further case–control studies are needed.

Our study had two limitations. The first was the small sample size. There were 30 cases of D‐ILD, of which only eight cases developed TAPO. Further studies are needed to determine which patients develop TAPO. Second, this study used a retrospective observational design. The interval between CT scans was unknown, which may have led to underestimation because all lung opacities, including TAPO, were not captured.

In conclusion, this study showed that grade 1 D‐ILD might include TAPO. Patients who develop TAPO can continue to receive osimertinib administration and may have better PFS.

When patchy opacities in the lung appear, it is important to consider the possibility of TAPO and to make an appropriate decision about osimertinib administration.

## AUTHOR CONTRIBUTIONS

MT contributed to the study conceptualization. KK, SF, RD, NI, KM, YH, and YK contributed to collect the data. CM and MT analyzed the data and wrote the article. All authors have approved the submitted version of the manuscript and agreed to be accountable for any part of the work.

## CONFLICT OF INTEREST

The authors have no conflicts of interest to disclose.
